# Locoregional control and survival after lymph node SBRT in oligometastatic disease

**DOI:** 10.1007/s10585-018-9922-x

**Published:** 2018-07-11

**Authors:** Mauro Loi, Michael Frelinghuysen, Natalie Desiree Klass, Esther Oomen-De Hoop, Patrick Vincent Granton, Joachim Aerts, Cornelis Verhoef, Joost Nuyttens

**Affiliations:** 1 Department of Radiation Oncology, Erasmus MC Cancer Institute, PO Box 2040, 3000 CA Rotterdam, The Netherlands; 2 Department of Pulmonary Medicine, Erasmus MC Cancer Institute, Rotterdam, The Netherlands; 3 Department of Surgical Oncology, Erasmus MC Cancer Institute, Rotterdam, The Netherlands

**Keywords:** Stereotactic body radiotherapy, Lymph node metastases, Oligometastases, Local therapy, Chemotherapy

## Abstract

Stereotactic body radiotherapy (SBRT) has emerged as an effective option in oligo-metastatic cancer patients affected by lymph node metastases, but its use might be questioned due to risk of regional and distant dissemination through the lymph node chain. The primary aim of our study was to assess the loco-regional control following SBRT in this setting. Ninety-one patients undergoing SBRT for at least one lymph node metastasis from miscellaneous primary tumors were retrospectively evaluated for patterns of failure and toxicity. locoregional relapse-free survival (LRRFS) and distant metastasis-free survival (DMFS) at 4 years were 79 and 44%. Repeated use of local therapy after progression resulted in a median interval of 17 months until allocation to systemic therapy or supportive care. Forty-three percent of patients were alive at 4 years. Local failure, occurring in 15% of patients, was the only predictor of poor survival (HR: 3.06). Tumor diameter ≥ 30 mm and urothelial primary tumor predicted for impaired local control (HR: 4.59 and 5.43, respectively). Metastases from pulmonary cancer showed a significant earlier distant dissemination (HR: 3.53). Only acute and late grade 1–2 toxicities were reported except for 1 case of G3 dysphagia. Loco-regional failure risk is low (18%) and justifies the use of local therapies for patients with oligometastatic disease. Durable disease remission can be achieved by iterative use of local approaches. Local control is correlated to improved OS. Diameter and primary tumor type may affect response to SBRT and risk for early metastatic dissemination.

**Electronic supplementary material:**

The online version of this article (10.1007/s10585-018-9922-x) contains supplementary material, which is available to authorized users.

## Introduction

Since the formulation of the concept of oligo-metastatic disease by Hellman in 2005, the existence of an intermediate condition between organ-confined and extensively disseminated malignancy has been debated. Indeed, loco-regional treatment by surgery or radiotherapy might be proposed to metastatic cancer patients with limited disease burden (3–5 metastases) [1] to avoid or postpone the use of chemotherapy [2]. This new paradigm of treatment integrated clinical practice in a number of different settings, such as limited liver or lung metastatic involvement from operated primary tumors [3–5], but it is unclear whether this policy could be beneficial in all cases. In particular, appropriate management of isolated lymph node metastasis often result in a dilemma for the clinician due to the high risk of subclinical dissemination along the node chain [6]. In recent years, stereotactic body radiotherapy (SBRT) emerged as a valuable option in the treatment of oligo-metastatic patients, delivering ablative radiation doses with limited toxicity, but the level of evidence for its applicability in the management of lymph node relapse is still poor. Initial reports showed satisfying local control rates with good to excellent survival, but there is a lack of information on the patterns of disease progression following SBRT [7]. Therefore, the primary aim of the study was to investigate the locoregional relapse-free survival. Secondary objectives were to investigate general outcome and predictors of disease control in a cohort of oligo-metastatic patients undergoing SBRT for lymph node metastases of treated primary tumors.

## Materials and methods

### Patient selection, treatment procedures and follow-up

Ninety-one consecutive patients with oligo-metastatic lymph node metastasis were treated at our Institution between May 2005 and September 2016 with stereotactic radiotherapy after curative treatment of the primary tumor. Indication to treatment was discussed at Multidisciplinary Tumor Board. Criteria for inclusion were oligo-metastatic lymph node metastasis with ≤ 3 metastases in two organs, considering the nodal drainage as one organ. Stereotactic treatment was performed using the Cyberknife radiotherapy system. Planning treatment volume (PTV) was obtained by isotropic expansion of 2–5 mm around the GTV (median 3 mm). The total dose was prescribed to the outer line of the PTV and was prescribed to the 75–85% isodose line using different fractionated schedules. Dose constraints for organs at risk (OARs) were applied according to the American Association of Physicists in Medicine (AAPM) recommendations [8]. Clinical evaluation with physical examination and CT-scan was performed at 3 and 6 months, and subsequently once a year until 5 years after the treatment or until disease progression. Assessment of efficacy and acute and late toxicity (within or after after 3 months from the end of treatment, respectively) was retrospectively performed using the revised response evaluation criteria in solid tumors (RECIST version 1.1, 2009) and the Common Terminology Criteria for Adverse Events (CTCAE version 4.03, 2010), respectively.

### Definition of the endpoints

Local failure (LF) was defined as an increase of at least 20% in diameter of the treated volume. Loco-regional failure (LRF) was defined as onset of one or more metastases in the same anatomical chain of the treated lymph node but outside the PTV. Distant metastases (DM) were defined as metastases occurring outside the anatomical chain of the treated lymph node. Any of the above cited events (LF, LRF, DM) when occurring for the first time after SBRT was considered as disease progression (DP). Local control (LC) was calculated from the start of radiation therapy to the date of LF. Loco-regional relapse-free survival (LRRFS), distant metastasis-free survival (DMFS) and disease-free survival (DFS) were measured from start of radiation therapy to occurrence of LRF, DM and DP, respectively. A corrected DFS (cDFS) was calculated as a surrogate indicator of chemotherapy-free survival as followed: local and/or distant progression effectively treated by a local curative approach such as surgery or SBRT was censored until the patient developed a new metastasis requiring systemic treatment or supportive care or until patient’s death. Overall survival (OS) was measured from start of the radiation therapy until death from any cause. Patients who did not experience any of the above-cited events were censored on the last day of contact.

### Statistical analysis

Statistical analysis was performed with IBM SPSS v.21 statistical software. We applied the Kaplan–Meier method to estimate survival curves and used the log-rank test to compare curves between levels of the following dichotomized categorical and continuous (cut-off: median value) variables: gender, age, primary tumor type (colorectal, lung, urothelial, cervical), use of 18-FDG-PET, diameter, biologically effective dose for α/β = 10 (BED), prior use of chemotherapy. A multivariable Cox regression analysis was performed including all risk factors with a p value of < 0.2 in the univariable analysis. Logistic regression was performed to test relationship between tumor diameter and LC. A p-value ≤ 0.05 was considered statistically significant.

## Results

### Baseline characteristics

Eighty-seven patients (96%) were referred with an isolated lymph node metastasis; four patients presented with a metastasis in a lymph-node and in another organ (brain, lung and liver in respectively 1, 1 and 2 patients). All patients underwent a Chest-Abdomen CT, while a restaging 18-FDG-PET was performed in 36 (39%) patients.

Thirty-five percent of these patients were previously treated with chemotherapy, 13% with surgery or SBRT for another metastasis, and in 52% no treatment had yet been given for the metastasis. Lymph node biopsy was collected in 48 patients (52%) while pathological diagnosis from a metastasis in another site was available in 14 out of 43 patients. Among the other 29 patients, nine underwent a restaging 18-FDG-PET following serum marker increase, while decision for treatment in the remaining 20 patients was based on progression on two CT scans due to technically infeasible procedure or refusal of the patient. Median diameter (defined as the maximum axial dimension as measured on planning CT scan) was 30 mm (5–63 mm). A fractionation scheme delivering a BED ≥ 75 Gy_10_ was used in 87% of patients.

Patient and treatment-related data are summarized in Table 1.

**Table 1:**
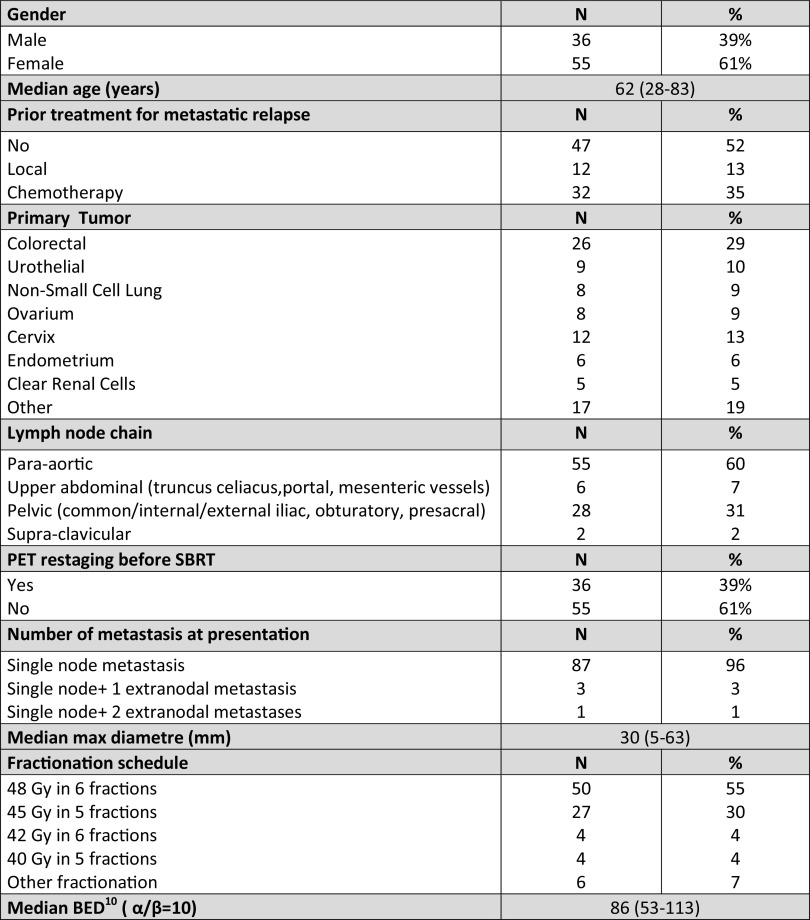
Clinico-pathological and treatment-related features of the study population

### Patterns of failure and treatment at progression

After a median follow-up of 23.3 months, 14 local failures, 16 locoregional relapses and 46 distant metastases were reported. At the time of our analysis, 56 patients experienced disease progression following SBRT: among them, disease progression was limited to ≤ 3 metastases in 32 patients, allowing for continuation of local treatments in 13 patients. Patterns of failure and treatment at progression are resumed in Table 2.

**Table 2:** Patterns of failure and treatment at secondary relapse following SBRT

Patterns of failure	N	Treatment at progression		
		BSC	Local treatment	Chemotherapy
Overall progression after SBRT	56 (62%)	13	15	28
≤ 3 metastases	32 (35%)	5	13	14
> 3 metastases	24 (26%)	8	2	14
Local progression	14 (15%)	5	2	7
Locoregional lymph node relapse	16 (17%)	4	4	8
Isolated relapse	3 (3%)	1	0	2
Concurrent distant metastases	13 (14%)	3	4	6
Distant metastases	46 (50%)	9	13	24

### Treatment outcome and patterns of care

Median follow-up after SBRT was 23.3 months (range 1.0–138.9, Inter-Quartile Range 9.9–42.3).

**Fig. 1:**
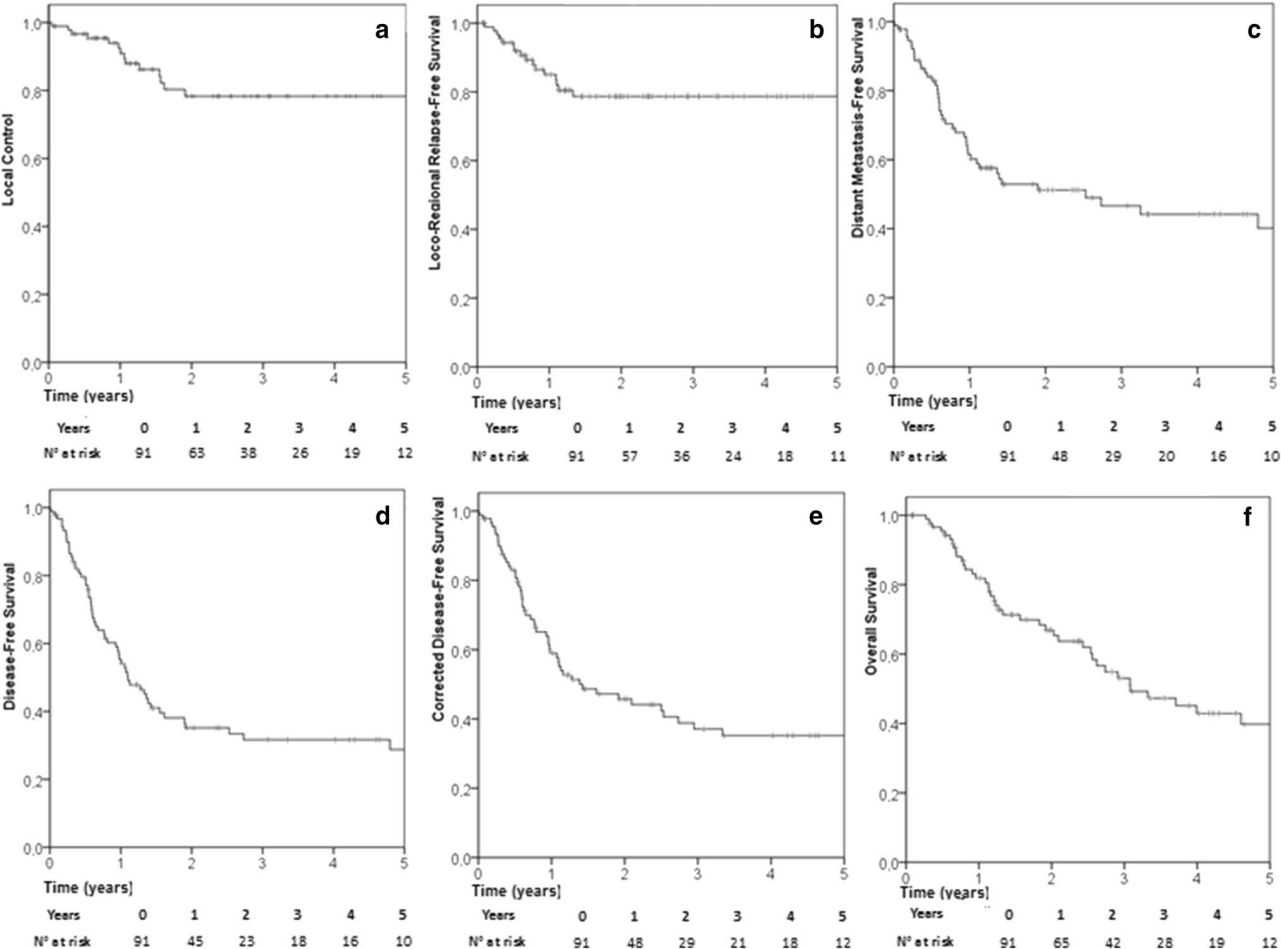
Kaplan Meyer curve (in years) for LC (**a**), LRRFS (**b**), DMFS (**c**), DFS (**d**), cDFS (**e**), and OS (**f**)

LRRFS was 79% at both 2 and 4 years (Fig. 1b): no predictive factor for relapse in neighboring lymph nodes was found at statistical analysis. Distant metastasis-free survival was 51% at 2 year and 44% at 4 years (Fig. 1c). Median time to metastatic failure was 26 months (95% CI 7–56). Primary lung cancer was the only predictor of impaired DMFS at both univariate (p = 0.015) and multivariate analysis (p = 0.02, HR: 2.65 [95% CI 1.17–6.02]).

**Fig. 2:**
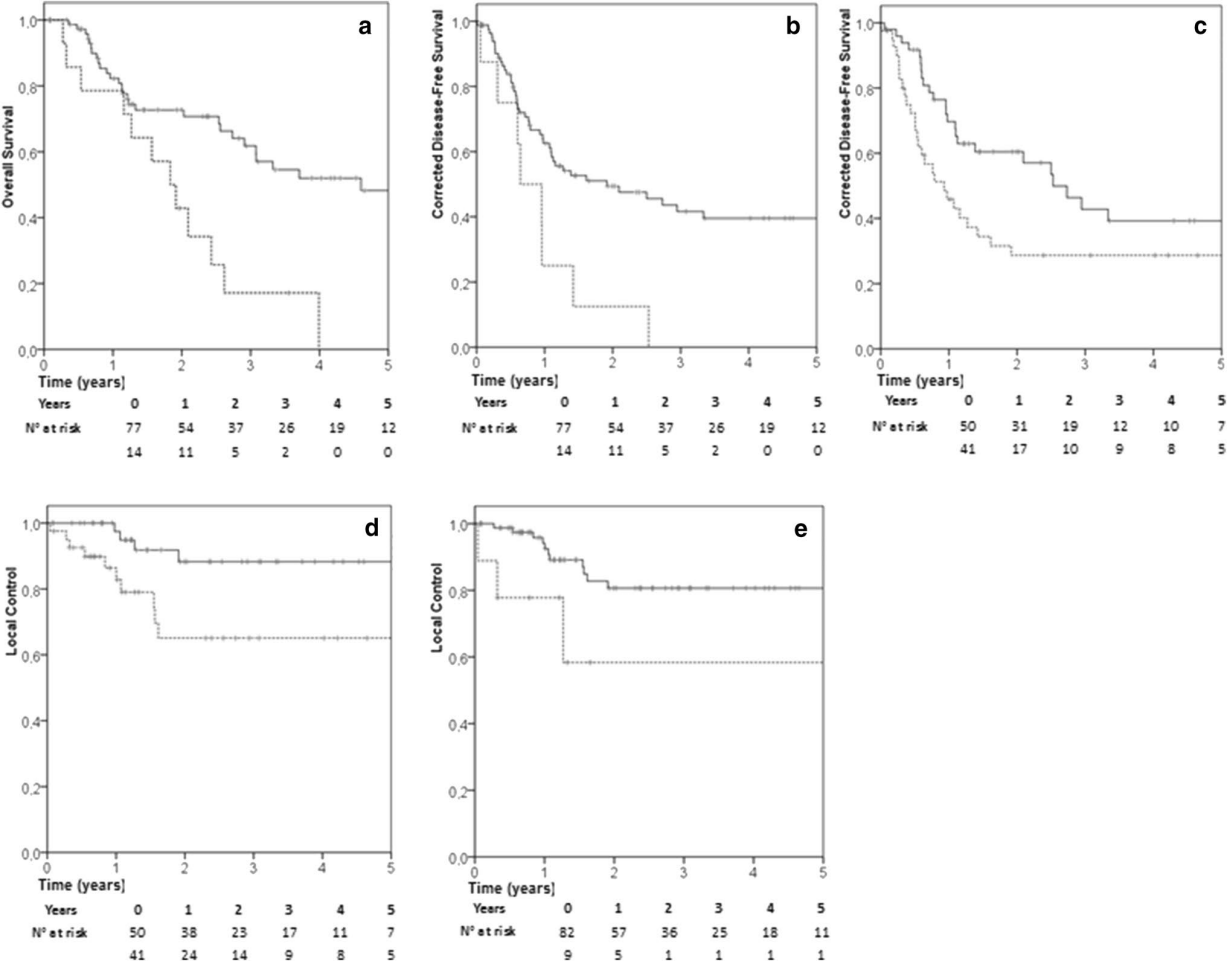
Kaplan Meyer curves for OS according to local failure (dashed line) versus local control (solid line), p = 0.001 (**a**); cDFS according to pulmonary (dashed line) versus non-pulmonary (solid line) primary, p = 0.01 (**b**); cDFS according to diameter ≥ 30 mm (dashed line) versus ≥ 30 (solid line), p = 0.02 (**c**); LC according to diameter ≥ 30 mm (dashed line) versus ≥ 30 (solid line), p < 0.001 (**d**); LC according to urothelial (dashed line) versus non-urothelial (solid line) primary p = 0.02 (**e**)

Two- and 4-years OS were 65 and 43%, respectively (Fig. 1f). Median overall survival was 36 months (95% CI 22–50). Lung primary, diameter ≥ 30 mm and LC were predictors of impaired survival at univariate analysis (p = 0.049, 0.029 and 0.001, respectively). However, only LC confirmed its predictive value at multivariate analysis (p 0.01, HR: 3.06 [95% CI 1.53–6.14]) (Fig. 2a).

DFS was 35% at 2 years and 31% at 4 years (Fig. 1e). Median time to disease progression was 13 months (95% CI 8–18). At univariate analysis, lung carcinoma (p = 0.006) and diameter ≥ 30 mm(p = 0.048) were significantly associated to disease progression. The predictive value of both lung primary and diameter (p = 0.01, HR: 2.69 [95% CI 1.27–5.73]; p = 0.048, HR: 1.71 [95% CI 1.00–2.90]) was confimed by multivariate analysis.

The 2 and 4-years cDFS were 46 and 35%, respectively (Fig. 1d). Median cDFS was 17 months (95% CI 5–28). Lung primary and diameter ≥ 30 mm were confirmed the main determinants of cDFS at both univariate (p = 0.01 and p = 0.02, respectively) and multivariate analysis (p = 0.01, HR: 2.59 [95% CI 1.21–5.57]; p = 0.02, HR: 1.71 [95% CI 1.10–3.26]) (Fig. 2b, c).

**Fig. 3:**
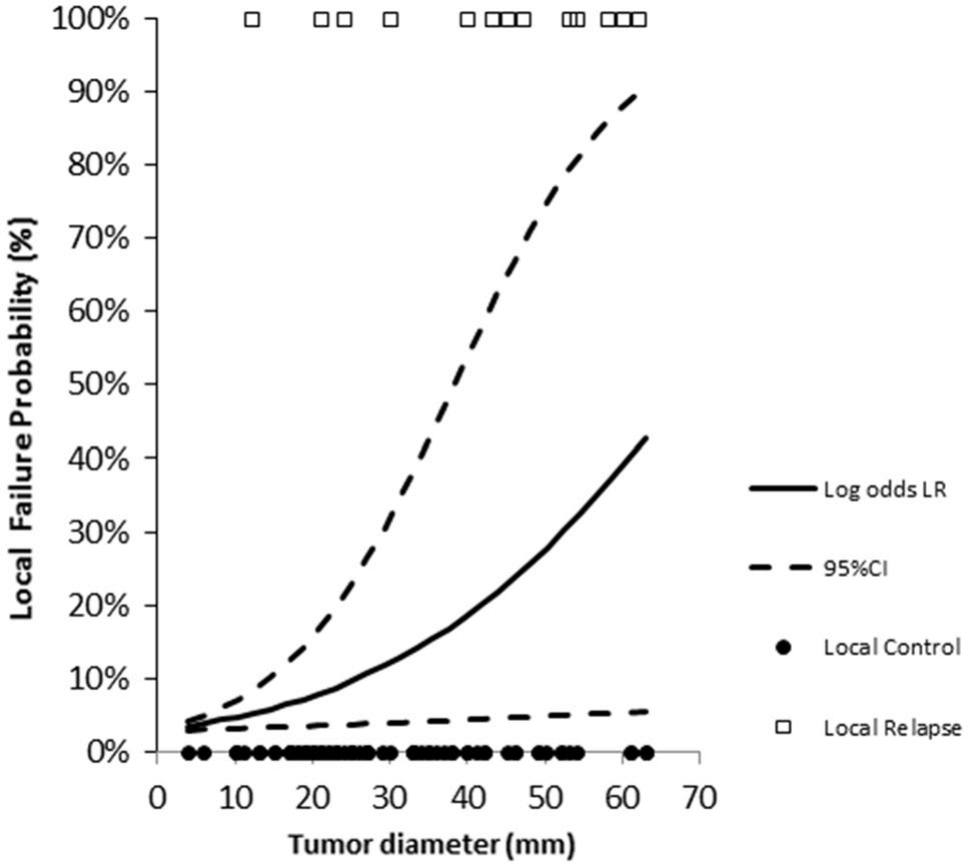
Solid line express the correlation between diameter (mm) and failure probability after SBRT. *Dashed line* 95% Confidence Interval. *Black dot* patients maintaining local control at the irradiated site. *White dots* patients experiencing local failure after SBRT

Concerning local control of the irradiated metastasis, 2 and 4-years LC was 78% (Fig. 1a). At univariate analysis, urothelial malignancy (p = 0.022) and diameter ≥ 30 mm (p = < 0.001) were correlated with impaired local control. Multivariate analysis confirmed that both urothelial origin (p = 0.015, HR: 5.43 [95% CI 1.42–20.84]) and diameter ≥ 30 mm (p = 0.013, HR: 4.59 [95% CI 1.41–14.95]) were independent risk factors for local failure (Fig. 2d-e). In a logistic regression model, a significant correlation between diameter and local failure was observed (p = 0.02) resulting in an increase in the odds for local failure failure by 1.053 [95% CI 1.011–1.096] for every 1 mm increase in diameter (Fig. 3).

### Treatment-related toxicity

Acute toxicity consisted mainly of mild Grade 1–2 nausea and diarrhea; exacerbation of pain and dysphagia were observed in a minority of patients. Symptoms required medical intervention in less than half of patients. Severe G3 dysphagia was reported in one patient that underwent feeding tube positioning during the SBRT course; symptoms spontaneously regressed after 1 week.

Focusing on late toxicity, no G3 toxicity was observed; the most common symptom following SBRT was mild G1-G2 chronic pain requiring no intervention or medication. No treatment-related death were observed in our cohort. Toxicities are reported in Table 3.

**Table 3:**
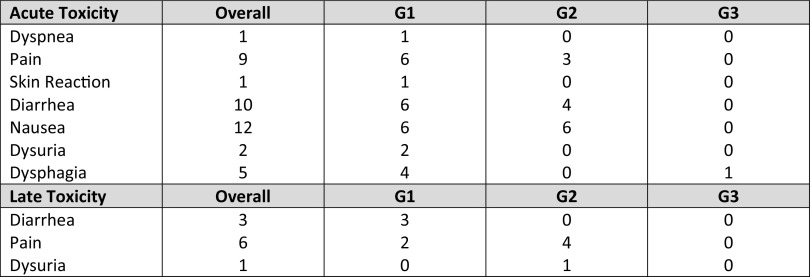
Acute and late toxicity

## Discussion

The hypothesis of our study is that focal treatment by SBRT in oligometastatic patients experiencing limited nodal relapse might be effective in arresting spread to the locoregional drainage and ultimately metastatic dissemination, irrespectively of patient baseline characteristics, prior treatments and primary tumor type. To test these hypothesis we reviewed a large consecutive serie of oligometastatic patients receiving SBRT for lymph nodal relapse after discussion at the Multidisciplinary Tumor Board for miscellaneous tumor types at different time points in the treatment sequence.

We reported the patterns of disease progression, in particular, early relapse to neighboring lymph nodes located on the same chain. This event occurred in the majority of patients at the same time of metastatic relapse to one or more extra-node districts, thus conferring theoretically no additional benefit to a more aggressive alternative approach encompassing the whole lymphatic drainage. On the other hand, selected patients who ultimately experience progression in proximity of the treated metastasis could still benefit from repeated SBRT or surgery in 25%. Therefore, onset of loco-regional relapse should not be considered as the result of inappropriate allocation to SBRT of patients that should have received upfront chemotherapy and should not influence the decision to perform further local therapies to prolong the systemic therapy free-interval, whenever feasible.

It is noteworthy that our report shows an impact of response to SBRT on survival in patients receiving SBRT for lymph node metastases, supporting the rationale of reducing the gross disease burden in oligo-metastatic patients by local approaches to improve outcome and alter the natural course of the disease. Most notably, after a median follow up of 23 months, 35% of patients did not experience any disease progression, suggesting that a curative perspective can be undertaken in selected patients in accordance with proposed modeling of oligo-metastatic disease [17]: future studies should identify this subset of long-lasting responders that might benefit from an exclusive local approach to maximize therapeutic ratio and avoid overtreatment.

In our study, SBRT resulted in durable disease remission, and its use in a strategy encompassing repeated use of local treatments for limited-extent relapse allowed to postpone the administration of systemic therapy in 46% of the patients for 2 years. Analysis of patterns of failure showed the value of target diameter and primary tumor as predictors of early local and distant failure, providing elements for selection of candidate patients for SBRT in the event of oligo-metastatic node involvement.

Lymph node metastases from solid cancers are a frequent occurrence in extensive metastatic dissemination due to the propensity of cancer cells to spread through the lymphatic drainage, often determining in-transit metastases [6]. However, in case of limited or isolated metastatic lymph-node involvement, therapeutic decision should consider the risk of subclinical invasion of neighboring lymph-nodes. For this reason, treatment options in the event of isolated node metastasis from miscellaneous primary tumors traditionally encompass the entire drainage, ranging from surgical lymphadenectomy to upfront systemic treatment : irradiation of the node chain with or without boost to the macroscopic site of disease has also been proposed [10–14]. However, several limitations must be taken into account: efficacy of chemotherapy can be limited depending on the primary tumor; lymphadenectomy can be contra-indicated due to disease extent, involvement of critical structures and operative morbidity; node chain irradiation may result in over-irradiation of critical structures, chronic lymphedema, and suboptimal efficacy due to the impossibility to the deliver high doses to an extended volume [10–14]. Moreover, according to the oligo-metastatic model, cancer cell spread might occur on a time-dependent, step-wise fashion, thus limited management of the clinically evident metastasis might be sufficient to obtain durable disease control and might exert an effect on overall survival by reducing the amount of actively replicating cells in the metastatic niche [15].

For these reasons, stereotactic body radiotherapy (SBRT) limited to macroscopic lymphadenopathies has been investigated by a number of authors, reporting promising results in terms of 1-years local control > 90% [16–22], optimal symptom control [22], long systemic therapy-free interval [21] and the possibility to iteratively perform SBRT in case of limited secondary relapse [19, 23]. Nevertheless, toxicity reports and lack of data regarding distant failure and impact on survival, raised doubts about the efficacy of this strategy [24].

It is noteworthy that in our cohort, following SBRT, disease progression was limited to ≤ 3 metastases in 32 out of 56 patients. This resulted in a further local treatment in almost one-third of these 32 patients as is shown in Table 2. Other authors had similar results [19, 23]. However, this strategy has been applied only in a minority of patients who maintained an oligo-metastatic pattern after disease progression: therefore patients possibly eligible for further SBRT or surgery might have been prematurely allocated to chemotherapy, which means we probably underestimate the theoretical benefit of a strategy based on multiple local therapy courses.

Local control rates at 2 and 4 years were in line with previously published reports [16–21]. As previously described in liver oligometastases [25], the main determinant of local efficacy was axial diameter of the metastasis, that is a known surrogate for volume [26] : this information might provide an useful tool to select patients eligible for SBRT, since according to our logistic regression model the risk of failure increase up to one-third for a diameter > 50 mm, and stresses the importance of timing of SBRT in order to avoid treatment delays that could profit tumor growth. Conversely, no impact of prior treatment (and by consequence of the time of onset of metastases in the natural history of the disease) was observed.

No treatment schedule corresponded to an advantage in terms of local efficacy: it could be argued that the use of high BED^10^ ≥ 75 Gy in the majority of cases did not allow a comparison between patient groups [27].

Toxicity was acceptable, with only one G3 acute event. Hoyer et al. [28] reported a treatment-related death and two bowel perforations in a mixed cohort of patients treated for abdominal metastases using a three-fraction schedule: in our study the use of 5–6 fractions schedules, predominant para-aortic localization, frequent use of tracking devices and acceptance of rigid dose constraints to the OARs may have contributed to the low toxicity rate.

Our study suffers from the usual limitations of retrospective studies, in particular possible presence of confounders and recall bias in the retrospective collection of outcome and toxicity data. Stereotactic radiotherapy in our cohort was the upfront treatment for metastasis in 52% of cases. Previously published series reported the use of SBRT after a first-line systemic therapy in 38–80% of cases [16–21]: promising outcome data may be influenced by inclusion of patients at an early time point of cancer natural history. Despite significant correlation was found between tumor diameter and risk of local failure, our logistic regression model was based on a small number of events and deserve further validation on larger series. The limited sample and the heterogeneity of treatment at progression do not allow to identify an optimal treatment sequence, in particular for patients with specific tumor types at higher risk of local failure or early distant dissemination. Due to retrospective evaluation of toxicity, underestimation or under-reporting of adverse events might not be excluded.

## Conclusions

After SBRT for lymph node metastases, the locoregional relapse free survival was 79% at 4 years. The overall survival was high (43% at 4 years) and it was correlated to local control after SBRT. Local therapies such as surgery or SBRT to new metastasis diagnosed after the SBRT allowed to postpone systemic therapies for 2 years in 46% of the patients. Diameter and type of primary tumor were correlated with risk of local and distant failure and might be taken into account in the clinical decision to perform SBRT. Hence, our results provided useful data for designing future studies on appropriate selection of candidate patients for this approach.

## Electronic supplementary material

Below is the link to the electronic supplementary material.

Supplementary material 1 (DOC 58 KB)

## Abbreviations

LF: Local failure
LRF: Loco-regional failure
DM: Distant metastases
DP: Disease progression
LC: Local control
LRRFS: Loco-regional relapse-free survival
DMFS: Distant metastasis-free survival
DFS: Disease-free survival
OS: Overall survival
cDFS: Corrected disease-free survival
SBRT: Stereotactic body radiotherapy
OARs: Organs at risk
GTV: Gross tumor volume
PTV: Planned tumor volume
BED: Biologically effective dose
