# Supplementary material for: Locoregional control and survival after lymph node SBRT in oligometastatic disease
Source: Clin Exp Metastasis. 2018 Jul 11;35(7):625–33. doi: 10.1007/s10585-018-9922-x (PMC6209001; doi:10.1007/s10585-018-9922-x)
Supplement: Supplementary file 1 — Supplementary material 1 (DOC 58 KB) [file 10585_2018_9922_MOESM1_ESM.doc]

| ***Variables*** | ***N*** | **LC** | | **LRR** | | **DMFS** | | **DFS** | | **cDFS** | | **0S** | |
| --- | --- | --- | --- | --- | --- | --- | --- | --- | --- | --- | --- | --- | --- |
|  |  | *UV*  *(p-value)* | *MV*  *HR [CI95%]*  *(p-value)* | *UV*  *(p-value)* | *MV*  *HR [CI95%]*  *(p-value)* | *UV*  *(p-value)* | *MV*  *HR [CI95%]*  *(p-value)* | *UV*  *(p-value)* | *MV*  *HR [CI95%]*  *(p-value)* | *UV*  *(p-value)* | *MV*  *HR [CI95%]*  *(p-value)* | *UV*  *(p-value)* | *MV*  *HR [CI95%]*  *(p-value)* |
| **Age**  **(in years)** | **≤62:** 46  **>62:** 45 | 0.3 |  | 0.7 |  | 0.15 | 0.6 [0.3-1.8]  0.1 | 0.21 |  | 0.49 |  | 0.59 |  |
| **Gender** | **Male**: 36  **Female**: 55 | 0.61 |  | 0.42 |  | 0.79 |  | 0.32 |  | 0.33 |  | 0.22 |  |
| **Pre-SBRT**  **PET staging** | **Yes:** 36  **No:** 55 | 0.72 |  | 0.36 |  | 0.31 |  | 0.39 |  | 0.21 |  | 0.79 |  |
| **Colorectal**  **Primary** | **Yes**: 26  **No:** 65 | 0.4 |  | 0.87 |  | 0.54 |  | 0.29 |  | 0.18 | 0.11 | 0.38 |  |
| **Cervical**  **Primary** | **Yes**: 12  **No:** 79 | 0.18 | 0.2 [0.1-3.3]  0.98 | 0.73 |  | 0.55 |  | 0.23 |  | 0.19 | 0.4 [0.2-1.2]  0.16 | 0.52 |  |
| **Urothelial**  **Primary** | **Yes**: 9  **No:** 82 | **0.02** | **5.4 [1.4-20.8]**  **0.02** | 0.24 |  | 0.39 |  | 0.49 |  | 0.85 |  | 0.36 |  |
| **Lung**  **Primary** | **Yes**: 8  **No:** 83 | 0.8 |  | 0.3 |  | **0.015** | **2.6 [1.2-6.0]**  **0.02** | **0.006** | **2.7 [1.3-5.7]**  **0.01** | **0.01** | **2.6 [1.2-5.6]**  **0.01** | **0.049** | 2.4 [0.9-6.1]  0.08 |
| **Diameter**  **(in cm)** | **<30:** 50  **≥30:** 41 | **0.001** | **4.6 [1-4-14.9]**  **0.01** | 0.95 |  | 0.21 |  | **0.048** | **1.7 [1.0-2.9]**  **0.03** | **0.02** | **1.7 [1.1-3.6]**  **0.02** | **0.029** | 1.6 [0.9-3.1]  0.12 |
| **Pelvic**  **Drainage** | **Yes:** 28  **No:** 63 | 0.32 |  | 0.25 |  | 0.26 |  | 0.38 |  | 0.27 |  | 0.97 |  |
| **BED_10_**  **(in Gy)** | **<86**: 39  **≥86**: 52 | 0.12 | 0.7 [0.2-2.8]  0.63 | 0.86 |  | 0.51 |  | 0.87 |  | 0.71 |  | 0.15 | 0.7 [0.4-1.4]  0.32 |
| **Prior Chemotherapy** | **Yes:** 32  **No:** 59 | 0.10 | 4.0 [0.7-14.0]  0.33 | 0.3 |  | 0.7 |  | 0.2 |  | 0.32 |  | 0.72 |  |
| **Local Recurrence** | **Yes:** 14  **No:** 67 | N/A |  | 0.79 |  | 0.1 | 3.1 [0.9-10.1]  0.07 | N/A |  | N/A |  | **0.001** | **3.1 [1.6-6.1]**  **0.01** |

Supplementary Table 1: Summary of univariated (UV) and multivariated (MV) analysis for variables correlated to LC (Local Control), LRRFS (Loco-Regional Relapse-Free Survival), DMFS (Distant Metastasis-Free Survival), DFS (Disease-Free Survival), cDFS (Corrected Disease-Free Survival), OS (Overall survival). N/A: not applicable. HR: Hazard Ratio.CI 95%: Confidence interval of 95%
